# Characterization of Anti-Insulin Antibodies in Type 1 and Type 2 Diabetes Mellitus: Clinical Relevance

**DOI:** 10.3390/ijms26041730

**Published:** 2025-02-18

**Authors:** Henrik Toft-Hansen, Christina Aniol-Nielsen, Daniel Elias, Madeleine Dahlbäck, Peter Rossing, Suvanjaa Sivalingam, William A. Hagopian, Darius A. Schneider, Claus H. Nielsen, Helene Solberg

**Affiliations:** 1 Non-Clinical and Clinical Assay Sciences, Novo Nordisk A/S, DK-2760 Maaloev, Denmark; 2 Centre for Functional Assays and Screening, Novo Nordisk A/S, DK-2760 Maaloev, Denmark; 3 Steno Diabetes Center Copenhagen, DK-2730 Herlev, Denmark; 4 Department of Clinical Medicine, University of Copenhagen, DK-2200 Copenhagen, Denmark; 5 Pacific Northwest Research Institute, Seattle, WA 98122, USA; 6 Institute for Inflammation Research, Center for Rheumatology and Spine Diseases, Copenhagen University Hospital, Rigshospitalet, DK-2100 Copenhagen, Denmark

**Keywords:** diabetes, anti-insulin antibodies, immunogenicity, neutralizing effect, glycemic control

## Abstract

The administration of insulin as a treatment for diabetes frequently leads to the formation of anti-insulin antibodies (IAs). The influence of these antibodies on the efficacy and safety of insulin therapy remains incompletely understood. This study presents a systematic, exploratory, cross-sectional analysis of the quantitative and qualitative properties of IAs in 101 patients with type 1 diabetes (T1D) and 101 patients with type 2 diabetes (T2D). The goal was to identify subpopulations of IAs that might impact glycemic control. We assessed the presence, titer, isotype, subclass, avidity, and in vitro neutralizing capacities of IAs, using glycated hemoglobin A1c (HbA1c) levels as an indicator of the clinical effectiveness of insulin. Our findings showed that 72% of individuals with T1D and 32% with T2D developed IAs, with IgG being the predominant isotype in both groups. Despite the presence of IAs, no in vitro neutralizing effect against insulin was observed, and there was no significant correlation between IA titer or avidity and HbA1c levels in either group. The results from this study demonstrate that while IAs are prevalent in both T1D and T2D, they do not have a significant clinical impact on the outcomes of insulin therapy in our study populations.

## 1. Introduction

Diabetes mellitus, either as an autoimmune condition (type 1 diabetes, T1D) or a metabolic-inflammatory disorder (type 2 diabetes, T2D), affects millions globally [1]. Advances in diabetes drug development have significantly enhanced the quality of life for these individuals. However, insulin products, being biologics, carry the risk of inducing an unwanted immune response, resulting in the formation of anti-exogenous insulin antibodies (IAs) [2]. The potential clinical effects of these antibodies include the neutralization of insulin’s pharmacodynamics by inhibiting its binding to insulin receptors. This can jeopardize blood sugar management, potentially affecting lipid and protein metabolism and causing long-term complications such as cardiovascular diseases, nephropathy, retinopathy, and neuropathy [3,4]. Additionally, the binding of IAs to insulin may result in the formation of antigen–antibody complexes, which could alter insulin’s pharmacokinetic response by affecting its half-life [5,6]. In rare instances, IAs may trigger immune-mediated hypersensitivity reactions, posing safety risks to patients [7].

Most therapeutic proteins can stimulate the production of anti-drug antibodies (ADAs), which might impact drug efficacy and compromise patient safety [8]. While exogenously delivered insulin is known to often induce ADAs (in this case, IAs) [9,10], generally, no significant impact on its efficacy or safety has been observed [11]. However, some patients with IAs experience poor glycemic control despite adequate treatment [12]. Although the causes of this dysregulation are likely multifactorial, IAs could contribute to diminishing the clinical effectiveness of insulin.

Some case reports have linked the presence of IAs with clinical issues such as insulin resistance, daytime hyperglycemia, and recurrent nocturnal hypoglycemia [2,13,14]. A classic example is “insulin autoimmune syndrome”, where hypoglycemia occurs in non-diabetic individuals due to autoantibodies against endogenous insulin [13]. Recently, the antibody response to exogenous insulin and its related clinical implications have been described as exogenous insulin antibody syndrome [15].

We hypothesized that a subpopulation of IAs might contribute to the glycemic dysregulation observed in some diabetes patients. This study aims to investigate whether differences in the properties of IAs (such as IA titer, isotypes, avidity, and in vitro neutralizing effects) can be identified in T1D and T2D patients receiving exogenous insulin therapy but exhibiting varying levels of glycemic control.

## 2. Results

### 2.1. Clinical and Demographic Characteristics of the Study Population

Demographic and clinical characteristics of the T1D and T2D study populations are presented in Table 1. Two hundred and two patients were included in the study. Of these, 101 were patients with T1D and 101 with T2D. A total of 50 (49.5%) of the 101 T1D patients were female, and among the T2D patients, 34 (33.7%) of the 101 patients were female. Mean age of T1D patients was 48 (range 18–79), whereas the T2D patients were markedly older with mean age of 67 (range 34–95). A total of 51 of the T1D and 47 of T2D patients had well-controlled blood glucose with maximum HbA1C levels of 53mmol/L at the time of screening. The remaining (50 in the T1D and 54 in the T2D group, respectively) patients had poorly controlled diabetes with HbA1C levels greater or equal to 69 mmol/L at screening. All patients included in the study were on insulin treatment at the time of sampling. Clinical characteristics of individuals with and without anti-insulin antibodies are presented in Table 2.

### 2.2. Prevalence and Titers of Insulin Antibodies and Glycemic Control

**Figure 1:**
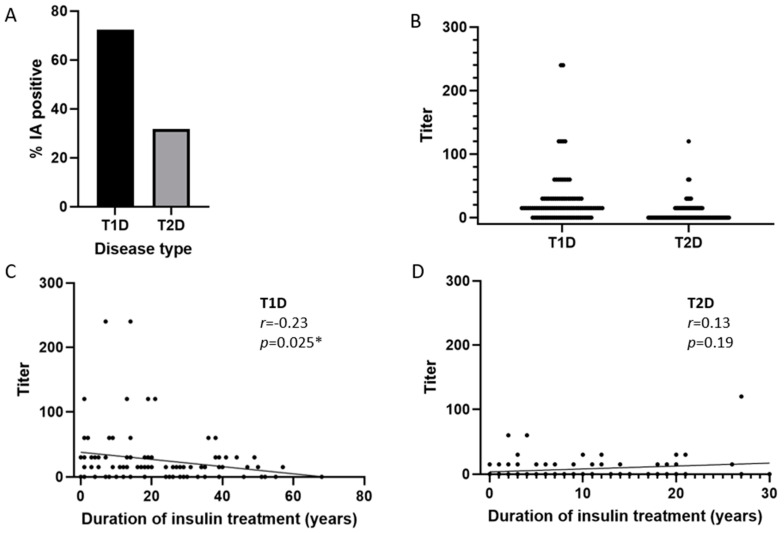
Prevalence (**A**) and titers (**B**) of insulin antibodies in patients with T1D and T2D. (**C**,**D**) show correlation analysis between IA titer and duration of treatment in T1D (**C**) and T2D (**D**). *r* and *p* values were derived from Spearman’s ranked analysis. IA: anti-insulin antibodies; T1D: type 1 diabetes; T2D: type 2 diabetes; Asterisk denotes statistical significance (*p*-value below 0.05).

A total of 72% of the patients with T1D and 32% of those with T2D were found to be positive for IAs in this study (Table 2 and Figure 1A). The median IA titer was 15 in both T1D and T2D (range: 15–240 in T1D and 15–120 in T2D) (Figure 1B). Analysis of the correlation between the titer of anti-insulin antibodies as a function of the duration of insulin treatment showed a weak but statistically significant negative correlation in T1D patients, but not in T2D patients over a period of up to 68 and 30 years, respectively (Figure 1C and Figure 1D).

**Figure 2:**
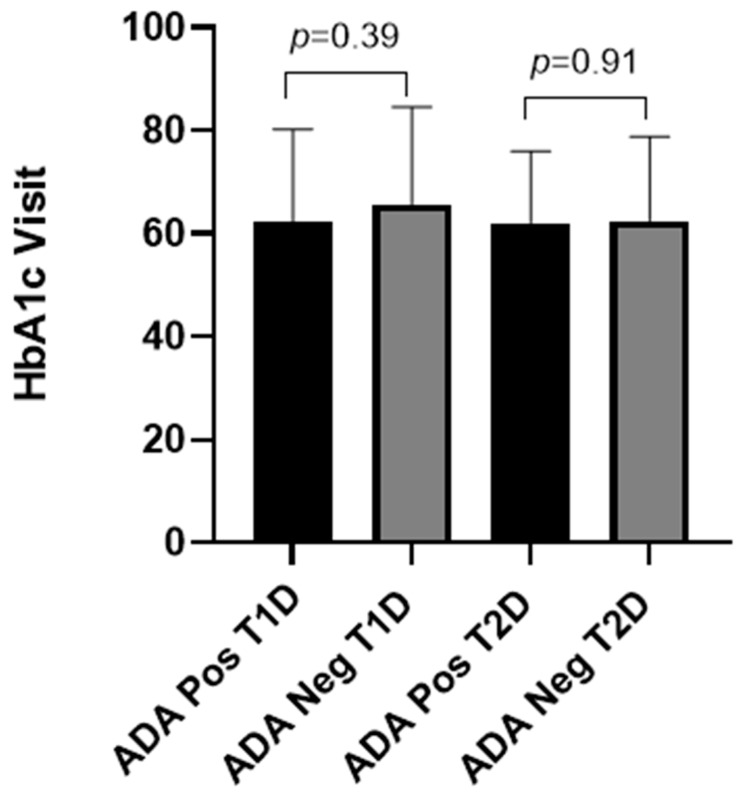
Levels of HbAc1 at Visit (mmol/mol) in T1D and T2D patients on insulin therapy with different IA status. *p*-values were calculated using two-tailed *t*-tests. HbA1c: hemoglobin A1c; ADA: anti-drug (insulin) antibody.

**Figure 3:**
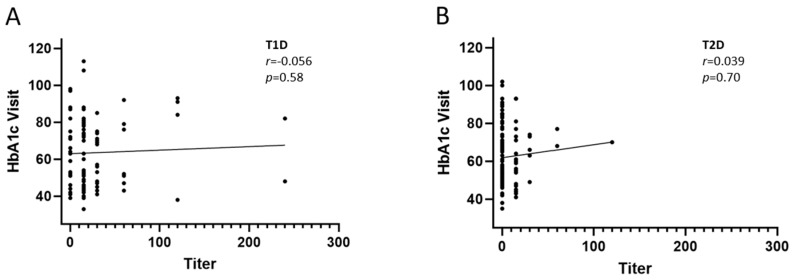
Correlation analysis of levels of HbAc1 at Visit (mmol/mol) in T1D and T2D patients on insulin therapy and titers of IAs in T1D (**A**) and T2D (**B**). *r* and *p*-values were derived from Spearman’s ranked analysis. HbA1c: hemoglobin A1c; T1D: type 1 diabetes; T2D: type 2 diabetes.

In this study, we used HbA1c levels at the sampling visit approximately 3 months after screening as a marker of glycemic control to assess whether the presence and/or titer of IAs correlate to HbA1c levels. The results showed that no statistically significant correlation between the presence of IAs and the HbA1C levels (Visit), either in T1D or in T2D (Figure 2). Next, we evaluated whether the level of IAs may have an impact on long-term glycemic control by evaluating the correlation between IA titer and HbA1c levels in patients that were positive for IAs. The results showed that no statistically significant correlation was observed between IA titer and HbA1c levels in T1D or T2D (Figure 3).

### 2.3. Avidity of Insulin Antibodies and Glycemic Control

**Figure 4:**
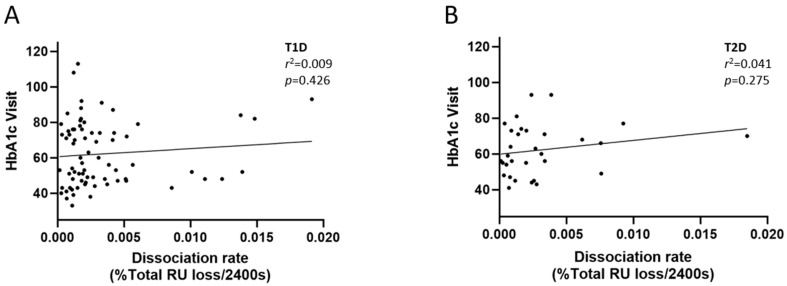
Correlation of HbA1c (a marker of glycemic control) and the dissociation constant of IAs as measured using Surface Plasmon Resonance analysis in both T1D (**A**) and T2D (**B**) as a measure of antibody avidity. *r*^2^ and *p*-values were derived from simple linear regression analysis. HbA1c: hemoglobin A1c; T1D: type 1 diabetes; T2D: type 2 diabetes, RU: resonance units.

The functional role of antibodies is influenced by the avidity of the antibodies to their respective antigens. To assess whether IA avidity has an impact on the clinical outcome of insulin treatment, we evaluated the dissociation rate of the IAs in patient sera using the Biacore^TM^ system as a measure of antibody avidity. We then correlated the dissociation rate to the long-term glycemic control as measured by levels of HbA1C. The result showed that there was no significant correlation between the antigen–antibody dissociation rates and the long-term glycemic control in both groups of patients (Figure 4).

### 2.4. Isotypes and Subclasses of Insulin Antibodies

**Figure 5:**
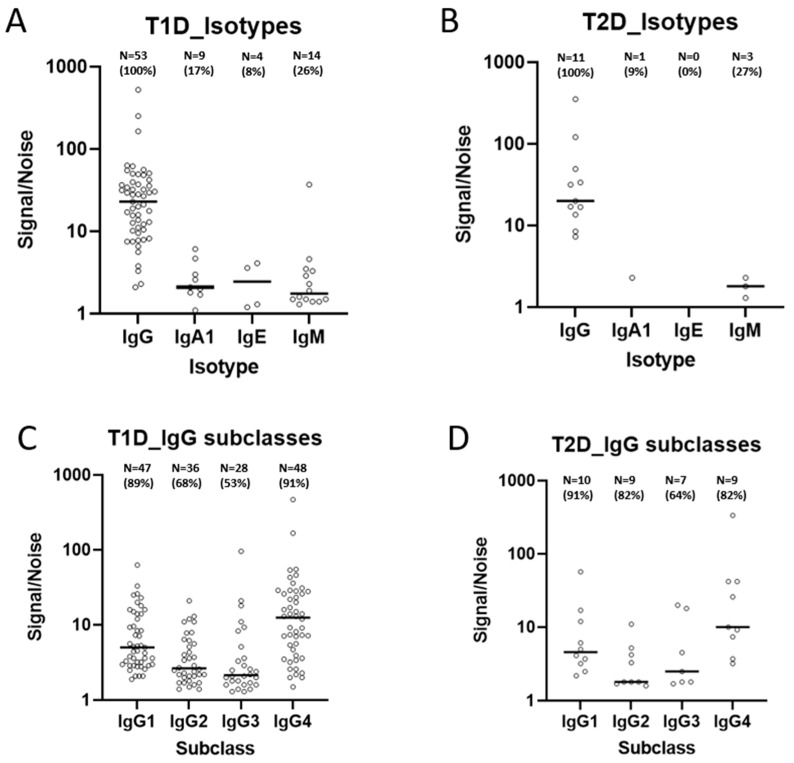
Isotype distribution of IAs in both T1D (**A**) and T2D (**B**) patients as expressed as signal-to-noise ratio. A ratio above 1 is defined as positive. The S/N values from individual isotypes and subclasses are not directly comparable. Isotypes were measured in 53 IA-positive T1D and 11 IA-positive T2D patients. In addition, IA IgG subclasses were evaluated in both T1D (**C**) and T2D (**D**) patients. In panels A and B, the signal-to-noise ratio for IgG was not measured directly but is presented as the sum of the individual IgG subclasses for each subject shown in panels (**C**,**D**), respectively. T1D: type 1 diabetes; T2D: type 2 diabetes; ADA: anti-drug (insulin) antibody; Ig: immunoglobulin.

**Figure 6:**
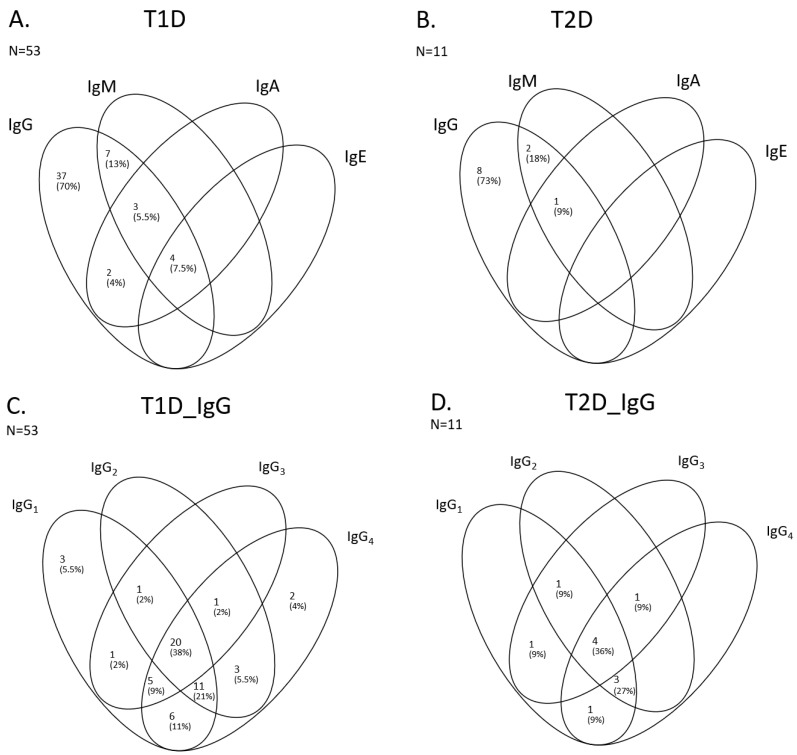
Venn diagrams show the number of patients that tested positive for the different antibody isotypes (**A**,**B**) and the IgG subclasses (**C**,**D**) in the T1D and T2D patient populations, respectively. T1D: type 1 diabetes; T2D: type 2 diabetes; Ig: immunoglobulin.

To further characterize the antibody responses to insulin and to examine its relevance to clinical outcome, we wanted to determine the isotype and subclass profiles of IAs in patients positive for IAs (Figure 5 and Figure 6). Isotype analysis was possible to perform in samples from 53 T1D patients and 11 TD2 patients positive for IAs, corresponding to approximately 60% of the IA-positive population. The results showed that the dominant antibody isotype was IgG, which was detected in all IA-positive patients in both T1D and T2D patients for whom isotype results could be obtained. The second most common IA isotype was immunoglobulin M (IgM), which was detected in 14 of 53 (26%) patients in T1D and in 3 of 11 (27%) of T2D patients tested for IA isotypes. IgA1 was the third most common IA isotype detected in 9 of the 53 (17%) T1D patients with positive IA and in 1 of the 11 (9%) IA-positive T2D patients. IgE was the least common IA identified with only 4 of the 53 IA-positive T1D patients (8%), and none of the 11 T2D IA-positive patients tested showed presence of IgE (Figure 5A,B). The distributions of IA isotypes are also presented in Venn diagrams (Figure 6A,B). The distributions of IA isotypes between T1D and T2D patients appeared similar.

Regarding IgG subclasses, IgG1 was detected in 47 of the 53 (89%) T1D patients tested for antibody subclasses, and in 10 of the 11 (91%) T2D patients that were positive for IA and were tested for antibody isotypes. IgG4 was detected in 48 (91%) of the 53 IA-positive T1D patients and in 9 of 11 (82%) T2D patients tested for IA subclass. IgG2 was detected in 36 of 53 (68%) T1D and in 9 of 11 (82%) of T2D patients tested for IA subclass. IgG3 was slightly less common compared to other IgG subclasses with a prevalence of 28 of 53 (53%) and 7 of 11 (64%) in T1D and T2D, respectively (Figure 5C and Figure 5D). The distributions of IA isotypes are also presented in Venn diagrams (Figure 6C,D). As for isotypes, there were no apparent difference in the distributions of IA IgG subclasses between T1D and T2D patients.

In the 53 IA-positive T1D patients tested for IA isotypes and subclasses, the proportion of patients who were in poor glycemic control (defined as HbA1c above 69 mmol/mol) at the sampling visit was 40%. We investigated the proportion of patients in that category for each IA isotype and subclass and did not identify any isotype or subclass where the proportion deviated substantially from 40%. Among T2D patients, the equivalent proportion was 64%, and no isotype or subclass deviated substantially from that.

### 2.5. Evaluation of the Neutralizing Activity of IAs

**Figure 7:**
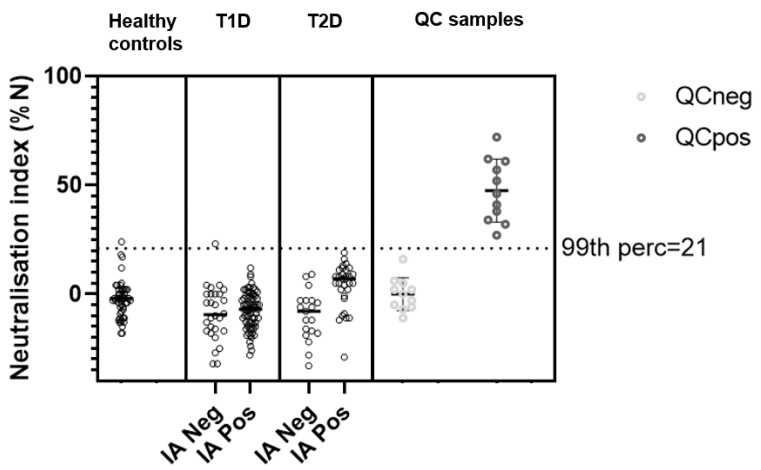
Neutralizing antibody levels measured in T1D and T2D patients as well as in healthy control subjects with no prior exposure to therapeutic insulin. Polyclonal antibodies raised in animals against therapeutic insulin were included as a positive control in the assay (QCpos). QCneg samples consist of a pool of normal healthy serum. The dotted horizontal line marks the 99th percentile result (21%N) for the healthy control samples. T1D: type 1 diabetes; T2D: type 2 diabetes; IA: anti-insulin antibodies; QC: quality control, perc: percentile.

To further characterize IAs in the study population, we evaluated the in vitro neutralizing activity of sera from patients that were positive for IAs. For comparison, serum samples from 50 healthy individuals were included as well as positive control samples. The results showed that none of the sera from IA-positive patients were positive for in vitro neutralizing antibodies (nAbs) against human insulin (Figure 7).

### 2.6. Evaluation of IA Titer and Insulin Dose

The interaction between IAs and insulin may lead to altered pharmacokinetics and pharmacodynamics, thereby affecting the insulin dose required for effective glycemic control. We therefore evaluated whether IA titer correlates to insulin dose in our study population, and the results showed no correlation between IA titer and the dose of insulin in our study population.

### 2.7. Effect of Concomitant Medication

A total of 7.9% of T1D and 74.3% of T2D patients used concomitant non-insulin anti-diabetic medications (primarily metformin and GLP-1 analogues) during the course of the study. To evaluate the potential confounding effects of such medications on the association between IAs and insulin-mediated long-term glycemic control, we performed a multivariate regression analysis. The results showed no indication of any confounding effect, and that the lack of correlation between IAs and insulin efficacy persists.

## 3. Discussion

This study provides valuable insights into the prevalence and characteristics of anti-insulin antibodies (IAs) in patients with type 1 diabetes (T1D) and type 2 diabetes (T2D) undergoing insulin therapy. The findings demonstrate that a significant proportion of these patients have circulating IAs, with 72% of T1D and 32% of T2D patients testing positive. These results align with previous studies indicating higher IA titers in T1D compared to T2D patients [9].

There are conflicting reports on the role of IA in the safety and efficacy of insulin. Reports have suggested that IAs may impact glycemic control [12,16,17]. Others have indicated that although IAs are common in patients on long-term insulin treatment, their relevance in glycemic control is not clinically relevant [18,19]. This indicates the need for further research to fully elucidate the relationship between anti-insulin antibodies and glycemic control.

In this study, we evaluated whether the presence and/or magnitude of IAs correlate with long-term glycemic control, as measured by the levels of Hb1Ac. No significant correlation was found between the presence or titers of IAs and long-term glycemic control. This is consistent with the reports of Achenbach et al. that showed that the presence of IAs was not associated with poor glycemic control, nor with the development of microvascular complications in individuals with T1D [18]. A more recent report by Chen et al. [20], who studied IA levels in patients with T2D, did not observe a measurable correlation between IA levels and blood glucose control [20]. Other factors, such as insulin resistance, beta-cell function, and medication adherence, seem likely to have a stronger influence on glycemic control than the presence of IAs [19]. While IAs did not correlate with long-term glycemic control, their presence may influence the short-term plasma glucose fluctuations. However, recent unpublished data from our group in another population of T2D patients demonstrated that the presence or titers of IAs did not show a significant correlation with self-measured plasma glucose levels (unpublished observation). This is in line with the report by Chen et al., who reported that IAs may be associated with local injection site reactions and total plasma insulin levels but have no effect on glycemic control or hypoglycemic episodes [20].

Notably, titers of IAs showed significant reductions with an increasing duration of insulin therapy in patients with T1D. The reason for this is not clear, but it may be that patients on long-term insulin treatments are older and have a poorer immune response. However, evaluation of the correlation between age and IA titer did not show a significant correlation , suggesting that a factor(s) other than age is at play in affecting IA titers. One possibility could be that the decrease in IA titers with increasing duration of insulin treatment is due to the development of immunological tolerance to insulin. This phenomenon has been observed in allergies and some autoimmune disorders such as multiple sclerosis [21,22], where repeated exposure to an antigen resulted in immunological tolerance. Whether long-term exposure to insulin leads to immunological tolerance to exogenous insulin remains to be addressed. Interestingly, the titers of IAs did not correlate to the duration of insulin therapy in T2D patients. Further studies are needed to fully elucidate the causes, mechanisms, and implications of reduced anti-insulin antibody generation in T1D over time.

Since the functional role of antibodies is dependent on their affinity to the antigen [23], we evaluated whether the avidity of IA correlates to the clinical impact of insulin by evaluating the IA dissociation constant, a measure of antibody avidity, using surface-plasmon resonance. The results showed that no significant correlation existed between the dissociation constant and the levels of HbA1c.

An interesting observation from our investigation of IA isotypes was that although most of the patients have been on long-term insulin therapy, anti-Insulin IgM antibodies were observed to be the second most common IA isotype. Considering that IgM is the type of response expected early during antigenic exposure, the high prevalence of IgM IAs can be considered surprising. Bohannon and colleagues have demonstrated that repeated antigenic stimulation can induce the development of long-lived IgM-producing plasma cells that may continue to produce IgM for a long time [24]. However, it remains to be seen whether exposure to exogenous insulin can induce the development of long-lived IgM-producing plasma cells.

Considering that antibodies of different isotypes differ in their effector responses [25], these differences may influence the clinical impact of insulin therapy [14]). We therefore evaluated the prevalence and distribution of IgG subclasses in our study population. A predominance of IgG1 and IgG3 may indicate stronger avidities and stronger effector responses whereas IgG2 and IgG4 may show weaker affinities and a milder immunological effector response [26,27]. Our results showed that IAs from T1D and T2D patients combined consisted of IgG, IgM, IgA1 and IgE antibodies. As expected, IgG is the most common antibody isotype observed in both groups of IA-positive patients. IgG subclass evaluation showed that IgG1 and IgG4 were the most common IA IgG subclasses followed by IgG2 and IgG3 is the least common of the IgG subclasses observed in our study patient populations. This finding is in line with a recent report by Chen and colleagues, who observed that T2D patients on long-term insulin treatment showed IgG1 and IgG4 as the dominant IgG subclasses [20]. Considering the relative abundance of IgG subclasses in the body, IgG4 is expected to be the least abundant subclass [28]. However, repeated exposure to an antigen may skew IgG responses in favor of IgG4 as a way to induce tolerance as reported by Uversky and colleagues [29], a subclass with reduced effector responses [28]. In the current study, no correlation was observed between IgG subtypes and the impact of insulin in glycemic control. However, the study’s cross-sectional design, lacking follow-up data to assess the effect of anti-insulin antibodies (IAs) over time, may have limited the scope of the study.

In our analysis, to investigate whether any IA isotype or subclass associated with a disproportionate risk of poorly controlled glycaemia, we did not identify an isotype or subclass, which appeared to make T1D or T2D patients prone to falling into the poorly controlled category at the sampling visit. Another contribution to the finding that IAs did not show a significant correlation to the clinical effect of insulin could be that the affinity of the antibodies towards insulin is lower than that of insulin binding to the insulin receptor. Neutralizing antibodies typically inhibit the biological activity of a drug by preventing it from binding to its target. However, if insulin binds more strongly to its receptor than the antibodies bind to insulin, the antibodies may not effectively prevent insulin from interacting with its receptor. Whether such an affinity difference is responsible for the lack of neutralization observed is a question that remains to be answered.

Indeed, evaluation of the in vitro neutralizing ability of IA antibodies showed that none of the serum samples from any of the two groups of patients showed the presence of antibodies with in vitro neutralizing ability. To the extent that the lack of in vitro neutralizing effect can be translated to the in vivo situation, this can help explain the lack of association between the presence of IAs and the clinical efficacy of insulin. To our knowledge, this is the first study in which in vitro neutralizing IAs have been evaluated in a clinical setting.

In summary, our data provide valuable insights into the prevalence and characteristics of anti-insulin antibodies (IAs) in individuals with type T1D or T2D undergoing insulin therapy. It provides detailed evaluation of IA isotypes and subclasses including avidity and potential impact on glycemic control.

## 4. Materials and Methods

Patients with either T1D or T2D who had been treated with insulin for varying durations were recruited from the outpatient clinic at the Steno Diabetes Center Copenhagen. At the time of study enrollment, participants were receiving insulin treatment for their respective conditions. The study included 101 patients from each group, totaling 202 participants, and was designed to include only those with either well-controlled glycemia, defined as an HbA1c level below 53 mmol/mol (7%), or poorly controlled glycemia, defined as an HbA1c level above 69 mmol/mol (8.5%). HbA1c levels were measured for each patient at the screening visit (referred to as “Screening”). Participants who met the criteria for one of the two glycemic control groups were invited to return approximately three months later for a sampling visit (referred to as “Visit”), during which a blood sample was collected for both HbA1c determination and IA analysis. Healthy controls (HCs) for this study were volunteer blood donors from Trina Bioreactives AG, Nänikon, Switzerland. The study was approved by the Danish Regional Scientific Ethics Committee, protocol no. H-17016539, and written informed consent was obtained from all participants that were included in the study.

### 4.1. Analyses of Insulin Antibodies and Titer

The samples were screened in a tiered approach for serum antibodies against human insulin (Novo Nordisk, Maaloev, Denmark) by radioimmunoassay (RIA), using an assay developed and validated according to current standards [30]. The samples were pre-treated with 300 mM Glycine-HCl pH 3.0 (*w*/*v*) to dissociate circulating insulin–IA immune complexes followed by precipitation of IA with PEG 6000 16%. The tracer was an equal mix of insulin labelled with I^125^ on one of four positions (A14, B16, B26, A19) through tyrosine-directed conjugation. The minimal required sample dilution (MRD) was 15. A screening cut point was set based on 50 HCs with a 5% false-positive rate. Subsequently, a confirmatory cut point was determined based on the same HCs with a 1% false-positive rate, by inhibiting the signal with an excessive amount of unlabeled insulin to the samples prior to analysis. We found similar cut points when sera from 50 insulin-naïve T2D individuals were tested in the assay. Assay set-up-specific cut points were normalized against the negative control serum pool, prepared from sera from 15 random HCs, using a floating cut point method. The assay sensitivity for IAs was determined using a pool (equal mix) of four mouse anti-human insulin monoclonal IgG antibodies HUI-001, HUI-018, OXI-005, and S1 (all from Novo Nordisk) as positive control anti-insulin antibodies. The screening assay sensitivity was determined to be 4 ng/mL using the control antibody mix, and the confirmatory assay had a sensitivity of 6 ng/mL. In the presence of 30 nM human insulin or 30 nM long-acting insulin analogue degludec (Tresiba^®^, Novo Nordisk), the screening assay sensitivity was 6 ng/mL and 50 ng/mL, respectively.

IA titers were determined by twofold titration of confirmed IA-positive samples. The titration cut point was set to 2times the normalized screening cut point, and the reported titers were multiplied by the MRD.

### 4.2. Dissociation Kinetics (Avidity Score)

To estimate antibody avidity, the kinetics of IA binding to insulin were analyzed directly in serum in a surface-plasmon resonance (SPR)-based assay on Biacore^TM^ systems (Cytiva, formerly GE Healthcare Life Sciences, Paris, France). Prior to analysis, the samples were filtered in Spin-X^®^ tubes (Corning^®^, Cornyn, NY, USA) by centrifugation and diluted 1:2 in HBS-EP+ running buffer (Cytiva, Marlborough, MA, USA). Human insulin (Novo Nordisk) was immobilized through primary amines on Series S biosensor CM5 chips (Cytiva) according to the manufacturer’s recommendations, to a concentration of 5 nM. The samples were then run over the chip at a fixed flow rate of 30 µL/min for 120 s and allowed to dissociate for 40 min. The chip was regenerated between each sample run with 10 mM Glycine-HCl, pH 1.5 followed by 50 mM NaOH, pH 12.7 (both Cytiva). A pool (equal mix) of mouse monoclonal anti-human insulin antibodies HUI-001, HUI-018, OXI-005; and S1 (all from Novo Nordisk) was used as the positive control.

The dissociation rates of IAs were evaluated from the observed percent loss in signal (resonance units, RUs) per second, which was evaluated from pre-defined report points on the binding sensorgrams. The observed values for percent RU loss were demonstrated to be independent of initial binding value.

### 4.3. IA Isotype and Subclass Determination

IA isotypes and subclasses were determined in a validated radiobinding assay (RBA), as described previously [31,32]. In brief, streptavidin resin beads (Fisher) were pre-incubated with biotinylated monoclonal antibodies (BD Pharmingen, San Diego, CA, USA or Southern Biotech, Birmingham, AL, USA) against the specific Ig classes. In a separate plate, the serum samples were pre-incubated with I^125^-labelled insulin (Tyr^A14^) (Perkin-Elmer, Springfield, IL, USA). Insulin-antibody immune complexes were then mixed with complexed beads for a secondary incubation. These final complexes were read using a scintillation counter as counts per minute (CPM). Isotype-specific cut offs were determined on each plate as follows: the mean of the negative control + 3 SDs. The negative and positive control sera for IgA1, IgM, and all IgG subclasses were kindly provided by William Hagopian, Pacific Northwest Research Institute (PNRI), Seattle, WA. The positive control for IgE antibodies was a mouse monoclonal anti-human insulin IgG antibody, HUI-001, that had been switched to a human IgE antibody backbone (Novo Nordisk). The results were reported as signal-to-noise (S/N) ratios.

### 4.4. Neutralizing Capacity

Samples identified as positive for insulin antibodies (IAs) by radioimmunoassay (RIA) were further analyzed for their in vitro neutralizing capacity, which refers to the ability to inhibit the interaction between insulin and its receptor. This was assessed using a commercially available cell-based functional assay (PathHunter^®^ Insulin Bioassay Kit, Eurofins DiscoverX, Fremont, CA, USA) optimized to detect neutralizing anti-insulin antibodies in serum samples. In this assay, when insulin binds to the receptors on the cells, the insulin receptor becomes phosphorylated. This phosphorylation activates the reporter enzyme β-galactosidase (β-gal) by facilitating the complementation of two inactive β-gal fragments into one active form through protein–protein interaction. The enzymatic activity of β-gal is then measured using a chemiluminescent substrate. The presence of neutralizing antibodies results in a decreased chemiluminescent signal due to reduced insulin receptor stimulation.

For the assay, serum samples and control sera were pre-incubated with human insulin (Novo Nordisk) and added to the cells. All subsequent steps followed the manufacturer’s protocol. A neutralizing cut point was established using serum from 50 healthy controls at a 1% false-positive rate, calculated using the 99th percentile (fixed cut point approach). The assay’s sensitivity was determined with a mouse monoclonal anti-human insulin IgG antibody, S1 (Novo Nordisk), and was found to be 400 ng/mL, with the capacity to tolerate at least 15 nM of the drug at a sensitivity of 565 ng/mL of antibody. The minimum required dilution (MRD) used was 10.

Results were reported as percent neutralization (%N), calculated using the following formula: %N = (1 − (unknown sample − non-stimulated sample)/(MAX − non-stimulated sample)) × 100%, where MAX represents a human serum pool sample (Trina Bioreactives AG) with 0.5 nM human insulin, and the non-stimulated sample is the same serum pool without human insulin. To inactivate complement, all serum samples were heated at 56 °C for 30 min prior to use.

### 4.5. Statistical Analyses

The statistical analyses were conducted to explore associations between IA characteristics and clinical outcome parameters. The specific statistical methods used are detailed in the relevant figure legends. All statistical calculations, analyses, and graphical representations were performed using GraphPad Prism, version9. For correlation analyses, R^2^ and *p* values were obtained from simple linear regression analysis. Other *p*-values were calculated using two-tailed *t*-tests.

## 5. Conclusions

In this study, we investigated the presence and properties of anti-insulin antibodies (IAs) in patients with T1D and T2D to determine if IAs and/or different isotypes and subclasses of IAs affect the efficacy of insulin therapy on glycemic control. The findings revealed that a majority of T1D and a third of T2D patients developed IAs, primarily of the IgG isotype. Despite the presence of IAs, no in vitro neutralizing effect against insulin was observed, and no significant correlation was observed between the presence of IAs, isotypes, or subtypes of the antibodies on long-term glycemic control, as measured by HbA1c levels, indicating that IAs do not significantly impact the efficacy of insulin therapy in our study population.

## Figures and Tables

**Table 1:** Clinical and demographic characteristics of the study population. Shown are the clinical parameters for all individuals included in the study, where available. The HbA1c values presented are from the screening visit, based on which the individuals were included in the study with HbA1c values ≤53 or ≥69 mmol/mol. GAD antibodies and C-pep were measured at earlier visits. BMI: body mass index; GAD: glutamic acid decarboxylase; C-pep: C-peptide; HbA1c: hemoglobin A1c; IU: international unit.

	Diabetes Mellitus Type	
	Type 1	Type 2
**Demography**		
Age (years) (mean, median, range)	(N = 101) 48, 48, 18–79	(N = 101) 67, 66, 34–95
Sex (F, M)	50, 51	34, 67
**Clinical parameters**		
BMI (mean, median, range)	(N = 100) 26.7, 26.0, 19.7–38.6	(N = 101) 30.9, 30.3, 18.5–46.9
GAD antibodies (U/mL) (mean, median, range)	(N = 69) 111, 69, 0–250	(N = 75) 13, 5, 0–250
C-pep (pmol/L) (mean, median, range)	(N = 87) 162, 30, 1.5–906	(N = 101) 1020, 821, 11–3496
Well-controlled glycemia at screening (HbA1c ≤ 53 mmol/mol) (mean, median HbA1c, range)	(N = 51) 47, 48, 35–52	(N = 47) 48, 48, 35–53
Poorly controlled glycemia at screening (HbA1c ≥ 69 mmol/mol) (mean, median HbA1c, range)	(N = 50) 82, 78.5, 70–112	(N = 54) 80, 78, 70–109
Age (years) at onset (mean, median, range)	(N = 101) 26, 24, 5–65	(N = 100) 49, 50, 27–83
Duration (years) of insulin treatment (mean, median, range)	(N = 100) 22, 19, <1–68	(N = 96) 9, 8, <1–30
Insulin dose (IU/kg) (mean, median, range)	(N = 82) 0.49, 0.48, 0.04–1.14	(N = 92) 0.76, 0.47, 0.05–13.37

**Table 2:** Clinical characteristics of individuals with and without anti-insulin antibodies. The HbA1c values presented are from the screening visit. GAD antibodies and C-pep were measured at earlier visits. DM: diabetes mellitus; ADA: anti-drug (insulin) antibody GAD: glutamic acid decarboxylase; C-pep: C-peptide; HbA1c: hemoglobin A1c; IU: international unit.

	DM Type 1 (N = 101)		DM Type 2 (N = 101)	
	ADA Positive	ADA Negative	ADA Positive	ADA Negative
**N (proportion)**	73 (72.3%)	28 (27.7%)	32 (31.7%)	69 (68.3%)
**Clinical parameters**				
GAD antibodies (U/mL) (mean, median, range)	(N = 47) 116, 76, 0–250	(N = 22) 99, 29, 0–250	(25) 22, 5, 0–250	(N = 50) 9, 5, 0–250
C-pep (pmol/L) (median, range)	(N = 62) 28, 1.5–616	(N = 25) 41, 1.5–906	(N = 32) 592, 11–3410	(N = 69) 944, 42–3496
Well-controlled glycemia (HbA1c ≤ 53 mmol/mol) (mean, median HbA1c, range)	(N = 38) 46, 48, 35–52	(N = 13) 48, 49, 42–52	(N = 15) 48, 49, 43–53	(N = 32) 47, 48, 35–53
Proportion of ADA pos/neg at Visit among well-controlled at Screening (N, %)	38/51 (74.5%)	13/51 (25.5%)	15/47 (31.9%)	32/47 (68.1%)
Poorly controlled glycemia (HbA1c ≥ 69 mmol/mol) (mean, median HbA1c, range)	(N = 35) 81, 76, 70–112	(N = 15) 86, 89, 71–100	(N = 17) 79, 76, 70–100	(N = 37) 81, 80, 70–109
Proportion of ADA pos/neg at Visit among poorly controlled at Screening (N, %)	35/50 (70%)	15/50 (30%)	17/54 (31.5%)	37/54 (68.5%)
Age at onset (median years, range)	(N = 73) 27, 5–65	(N = 28) 20.5, 6–46	(N = 32) 46.5, 30–79	(N = 68) 51, 27–83
Duration of insulin treatment (mean, median years, range)	(N = 72) 20, 18, 0–57	(N = 18) 26, 26, 0–68	(N = 30) 10, 10, 0–27	(n = 66) 8, 8, 0–30
Insulin dose (IU/kg) (mean, median, range)	(N = 58) 0.48, 0.46, 0.04–1.09	(N = 24) 0.52, 0.48, 0.10–1.14	(N = 30) 1.00, 0.49, 0.12–13.37	(N = 62) 0.64, 0.46, 0.05–4.72

## Data Availability

The data presented in this study are available on request from the corresponding author due to European Union General Data Protection Regulation (GDPR) to ensure data privacy of the subjects participating in this clinical research project.
